# Encouraged to Complete the Vaccination Articles

**Published:** 1898-07

**Authors:** Alex. Wheeler

**Affiliations:** 37 Victoria Road, Darlington, England


### ENCOURAGED TO COMPLETE THE VACCINATION ARTICLES.

37 VICTORIA ROAD, DARLINGTON, ENGLAND,
May 15th, 1898.

EDITOR:—I hope that the response to your request for opinions as to the Royal British Commission articles of Dr. Leverson has been such as to encourage you in their completion. I shall feel great sympathy with you under the ordeal if not.

I am yours truly,

ALEX. WHEELER.

